# Supplementary material for: Neurofunctional underpinnings of individual differences in visual episodic memory performance
Source: Nat Commun. 2023 Sep 14;14:5694. doi: 10.1038/s41467-023-41380-w (PMC10502056; doi:10.1038/s41467-023-41380-w)
Supplement: Supplementary file 1 — Supplementary Information [file 41467_2023_41380_MOESM1_ESM.pdf]

Supplementary Materials for

Neurofunctional underpinnings of individual differences in visual episodic memory performance

Léonie Geissmann*, David Coynel, Andreas Papassotiropoulos, Dominique J. F. de Quervain*

*Corresponding authors. Email: leonie.geissmann@unibas.ch or dominique.dequervain@unibas.ch

This PDF file includes:

Figs. S1 to S20
Tables S1 to S3
Methods S1

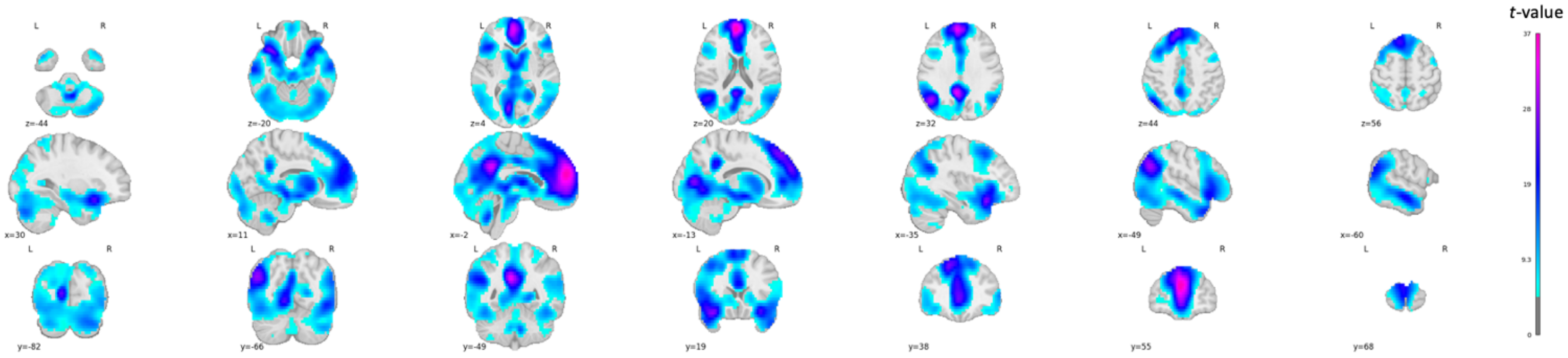

Fig. S1. Statistical brain map of the group-based positive subsequent memory effects. The images are corrected for multiple comparisons at the whole-brain level (two-sided *t*-test, *p*-FWE-corrected < 0.05, *t*-FWE-corrected = 4.848).

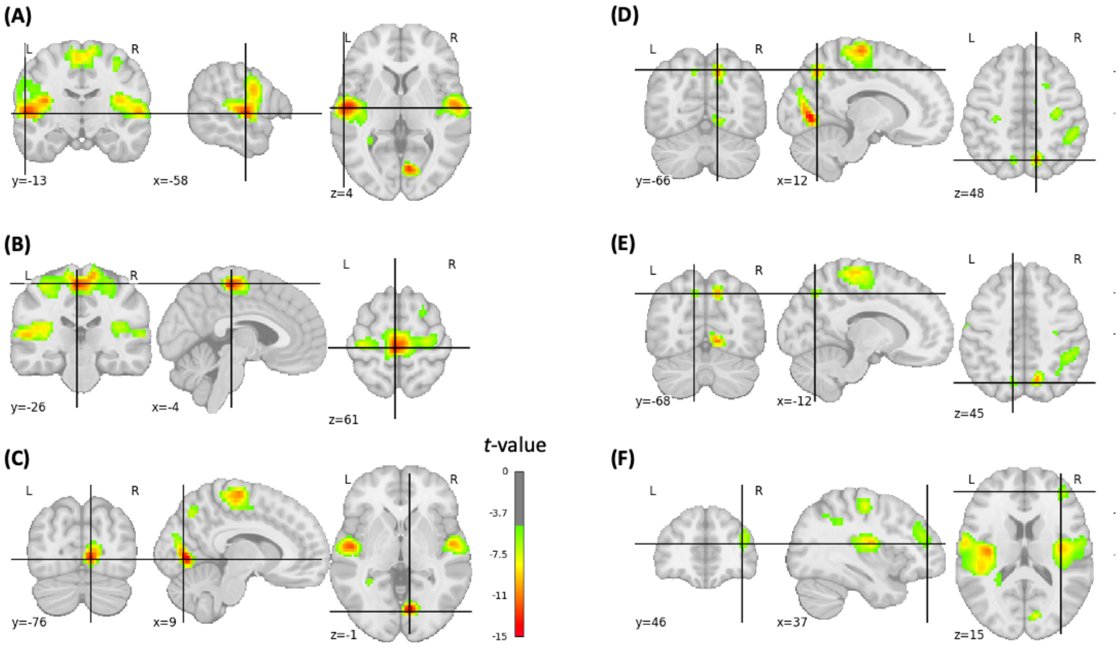

Fig. S2. Statistical brain map of the group-based negative subsequent memory effects. Coordinates were placed in the following regions: **(A)** Planum temporale/Heschl's gyrus (t = -12.82)*,* **(B)** precentral gyrus (t = -12.64), **(C)** lingual gyrus/intracalcarine gyrus (t = -13.45)*,* **(D)** precuneus (t = -10.73)*,* **(E)** precuneus (t = -6.85*),* **(F)** frontal pole (t = -5.63). The images are corrected for multiple comparisons at the whole-brain level (two-sided t-test , p-FWE-corrected < 0.05, t-FWE-corrected = -4.848).

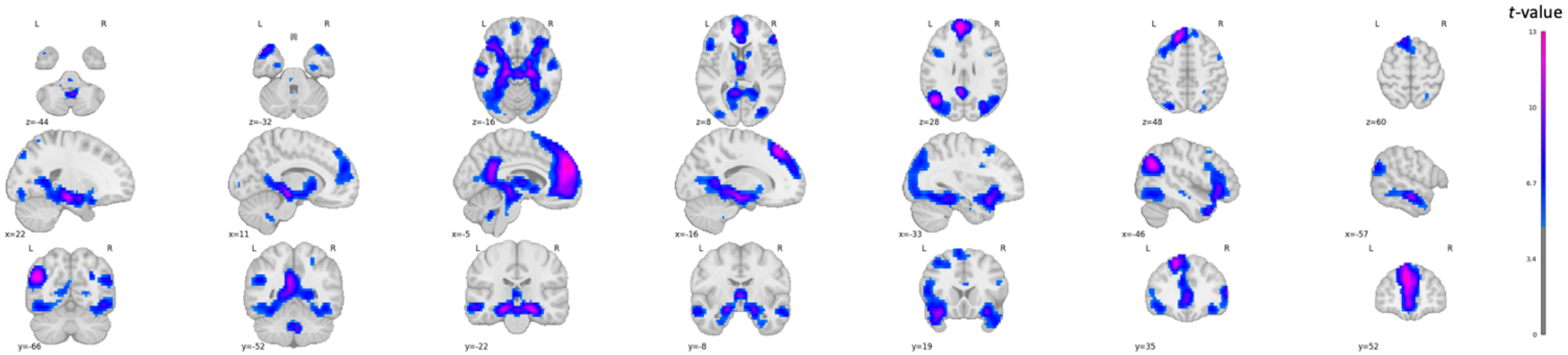

Fig. S3. Statistical brain map of the group-based memorability-controlled positive subsequent memory effects. The images are corrected for multiple comparisons at the whole-brain level (two-sided *t*-test, *p*-FWE-corrected < 0.05, *t*-FWE-corrected = 4.82).

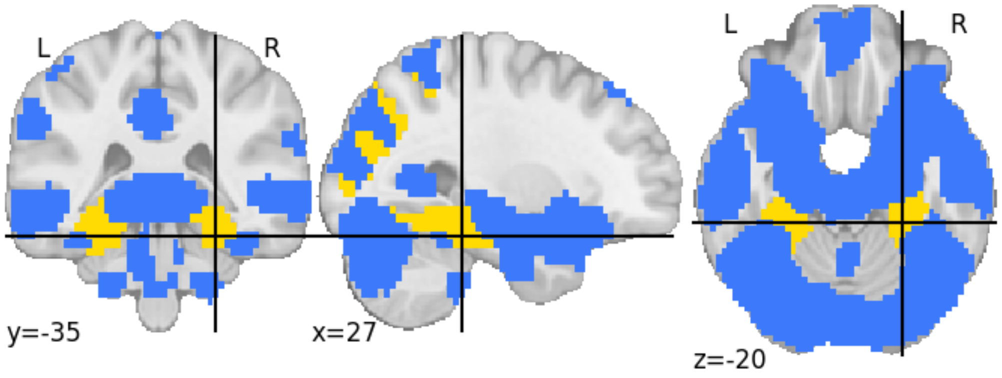

Fig. S4. Positive subsequent memory effects that emerged only when controlling for memorability. The yellow voxels, largely located in the fusiform gyrus, did not result from the classical subsequent memory effect analysis, which is here colored blue (two-sided p-FWE-corrected < 0.05 $t = 2.53$ versus $t = 9.35$ when not controlling and controlling for memorability, respectively, at the shown coordinate, both calculated with a two-sided t-test). The figure was created by overlaying the respective binarized statistical maps.

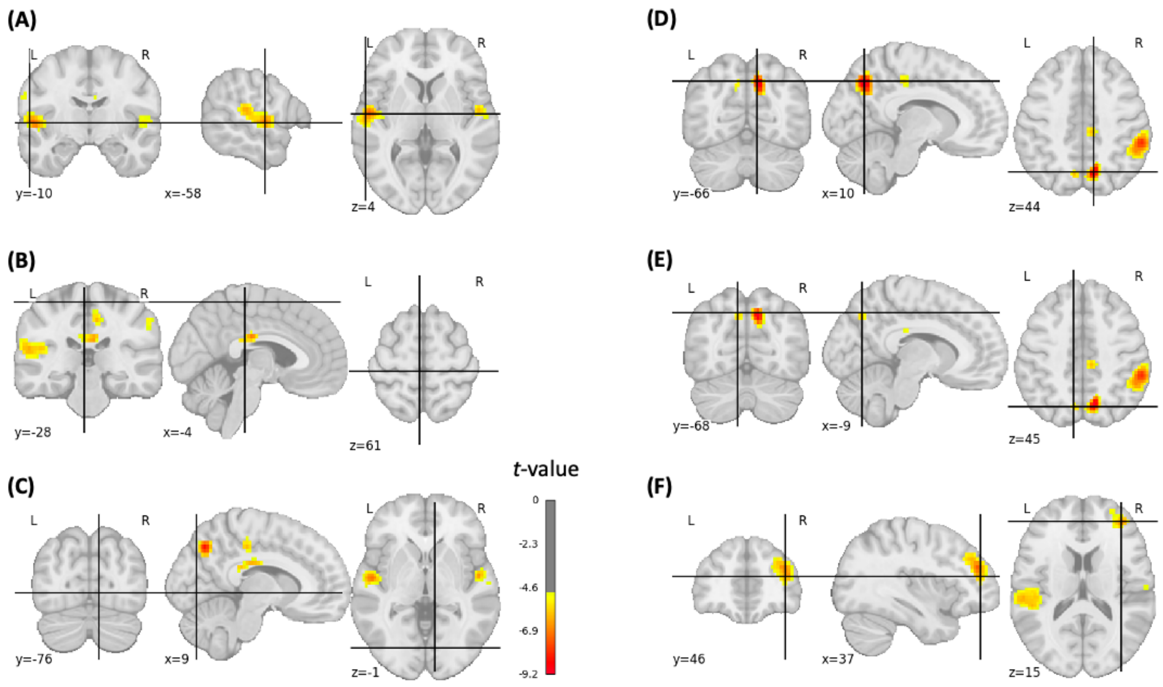

Fig. S5. Statistical brain map of the group-based memorability-controlled negative subsequent memory effects. Coordinates were placed alike Fig. S2: **(A)** Planum temporale/Heschl's gyrus (t = -7.09)*,* **(B)** precentral gyrus left (t = -3.38), **(C)** lingual gyrus/intracalcarine gyrus (t =-0.07*,* **(D)** precuneus right (t = -9.21)*,* **(E)** precuneus left (t = -6.03*),* **(F)** frontal pole right (t = -6.23). Images are corrected for multiple comparisons at the whole-brain level (two-sided *t*-test, *p*-FWE-corrected < 0.05, *t*-FWE-corrected = -4.82).

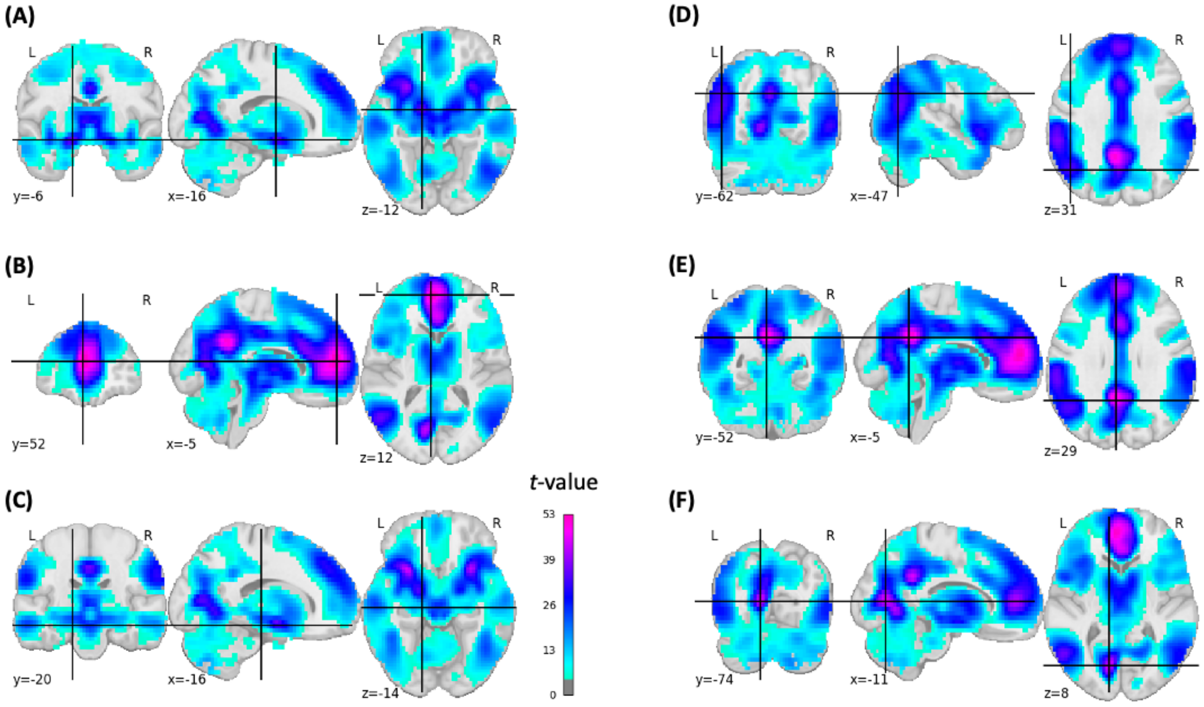

Fig. S6. Statistical brain map of the group-based positive memorability effects. For illustrative purposes, coordinates were placed in left-hemispheric brain regions: **(A)** amygdala (t = 35.89)*,* **(B)** caudal anterior cingulate (t = 52.59), **(C)** hippocampus (t = 13.18)*,* **(D)** superior lateral occipital cortex/angular gyrus (t = 33.17)*,* **(E)** PCC (t = 48.71*),* **(F)** pericalcarine cortex (t = 44.86). The images are corrected for multiple comparisons at the whole-brain level (two-sided t-test, p-FWE-corrected < 0.05, t-FWE-corrected = 4.849; see Methods S1).

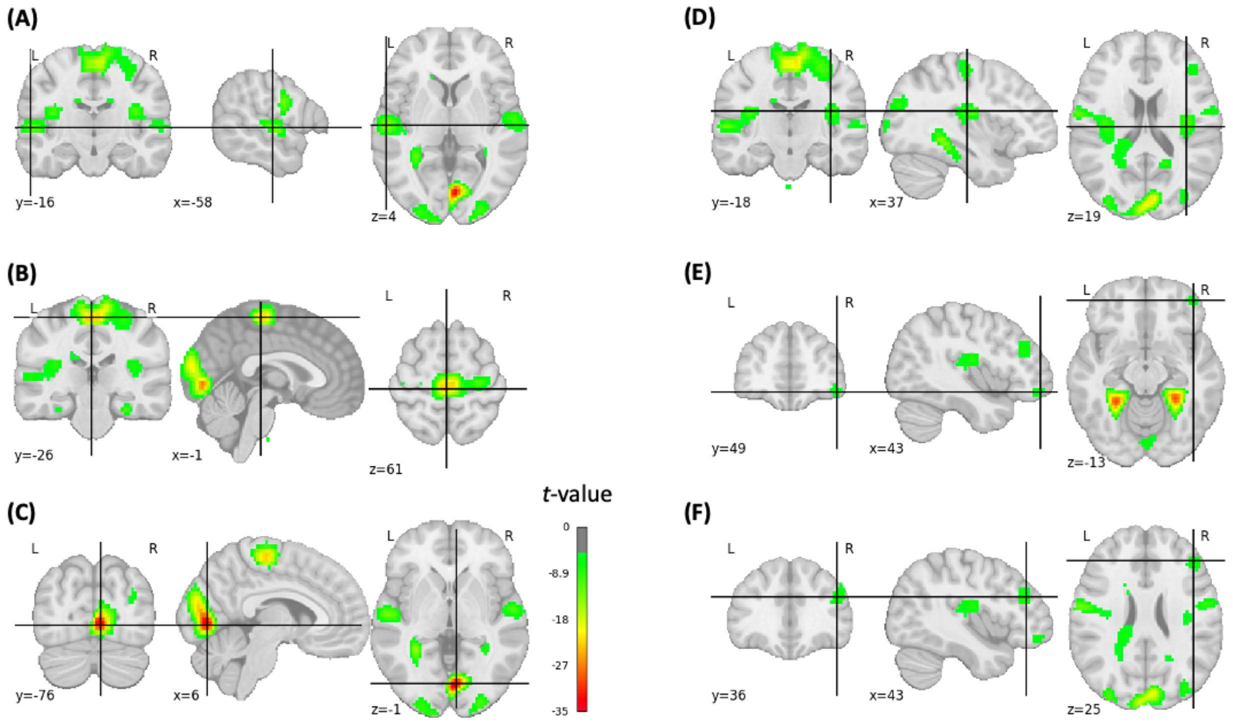

Fig. S7. Statistical brain maps of the group-based negative memorability effects. Coordinates were placed in the following brain regions: **(A)** planum temporale/Heschl's gyrus left (t = -13.44), **(B)** precentral gyrus left (t = -21.98), **(C)** lingual gyrus/intracalcarine gyrus right (t =-35.47), **(D)** central opercular cortex/insular cortex/parietal operculum cortex right (t = -9.64), **(E)** frontal pole right (t = -6.76), **(F)** middle frontal gyrus right (t = -8.14). The images are corrected for multiple comparisons at the whole-brain level (two-sided t-test, p-FWE-corrected < 0.05, t-FWE-corrected = -4.849).

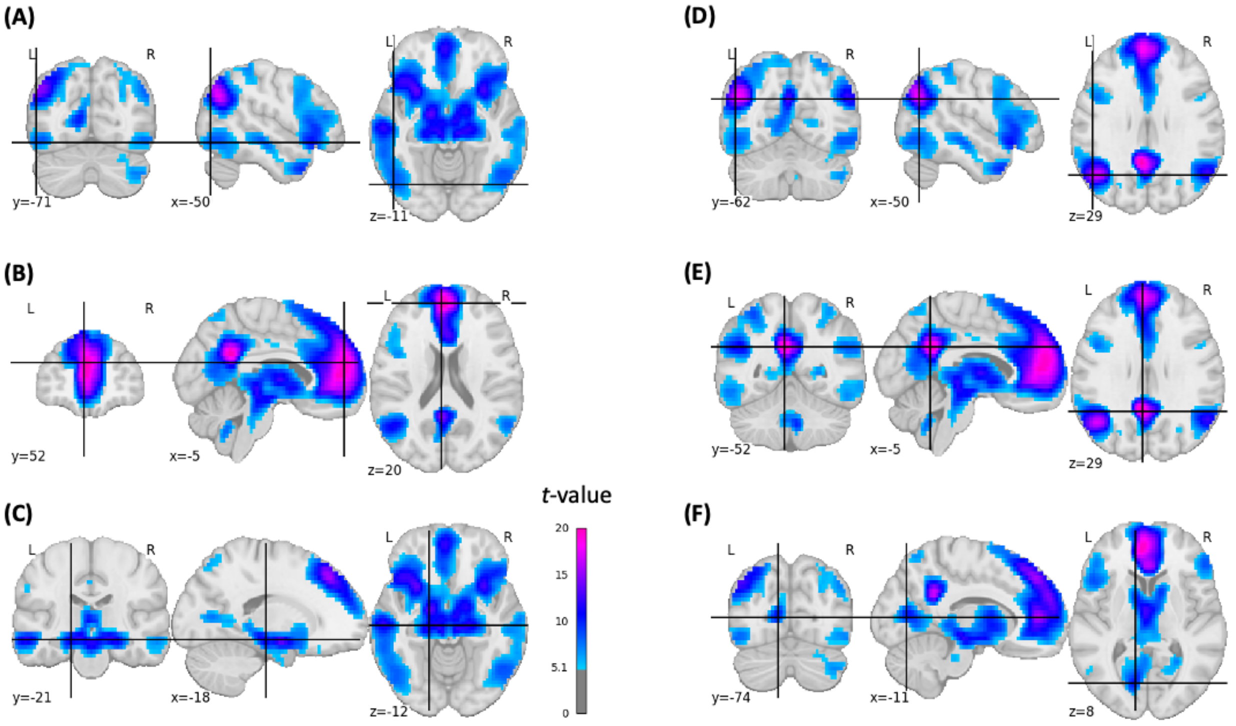

Fig. S8. Statistical brain map of the group-based arousal-controlled positive subsequent memory effects. For illustrative purposes, coordinates were placed in left-hemispheric brain regions: **(A)** inferior lateral occipital cortex (t = 8.19), **(B)** superior frontal gyrus (t = 19.57), **(C)** hippocampus (t = 11.18), **(D)** superior lateral occipital cortex/angular gyrus (t = 17.08), **(E)** PCC (t = 20.13), **(F)** intracalcarine cortex (t = 10.13). The images are corrected for multiple comparisons at the whole-brain level (two-sided t-test, p-FWE-corrected < 0.05, t-FWE-corrected = -4.836).

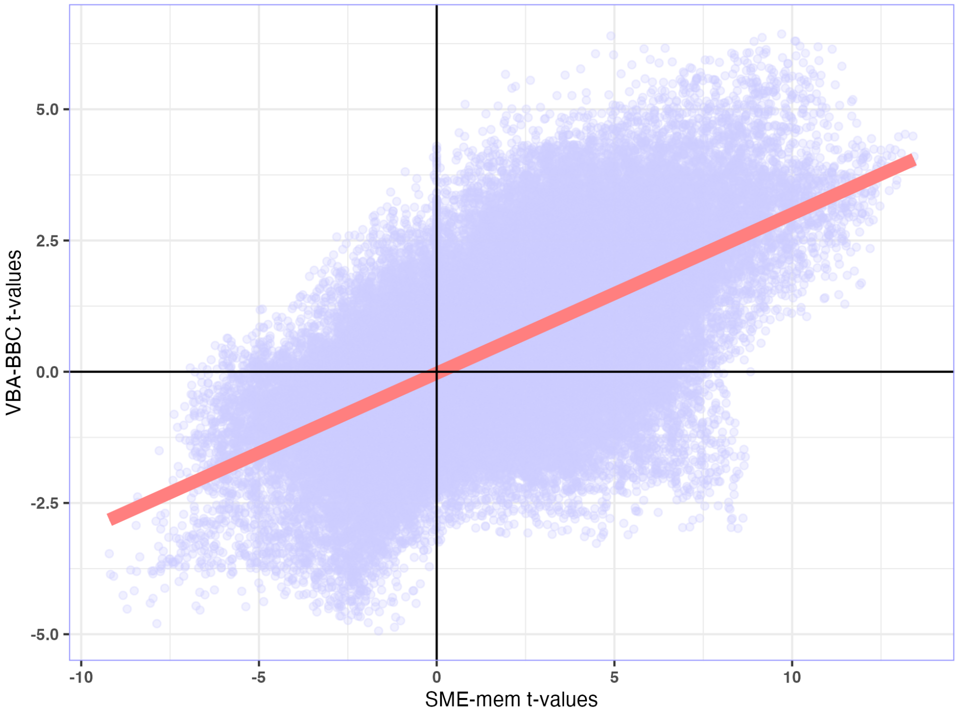

Fig. S9. Voxel-wise correlation of the *t*-values of the voxel-based brain-behavior correlations and the memorability-controlled subsequent memory effects. The data points represent the *t*-values of each voxel for both analyses, and the regression line demonstrates the positive association between the two ($t(72439) = 214.40$, p-uncorrected $< 2.2e\text{-}16$, $R^2 = 0.39$). A linear regression model was used. Abbreviations: SME-mem, subsequent memory effects controlled for memorability; VBA-BBC, voxel-based approach brain-behavior correlations.

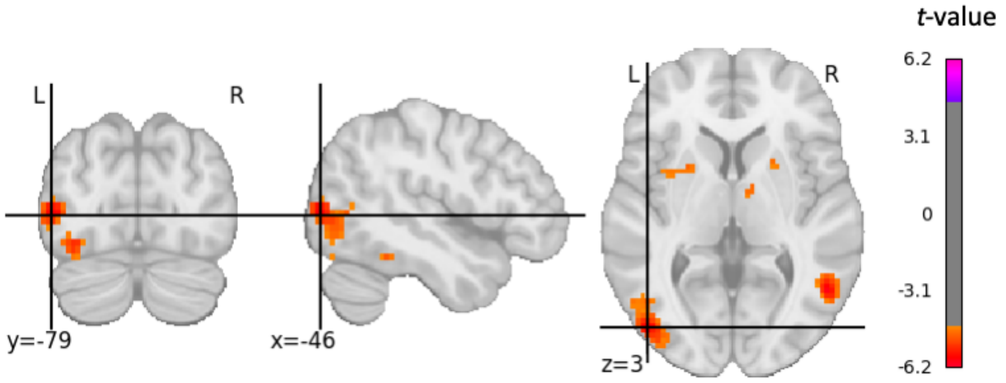

Fig. S10. Inter-individual correlations between individual subsequent memory effects and free recall performance using a voxel-based approach. Coordinates were placed in the lateral occipital cortex (t = -5.86)**. The images are corrected for multiple comparisons at the whole-brain level (two-sided *t*-test, *p*-FWE-corrected** < 0.05, t-FWE-corrected = |4.538|).

(A)

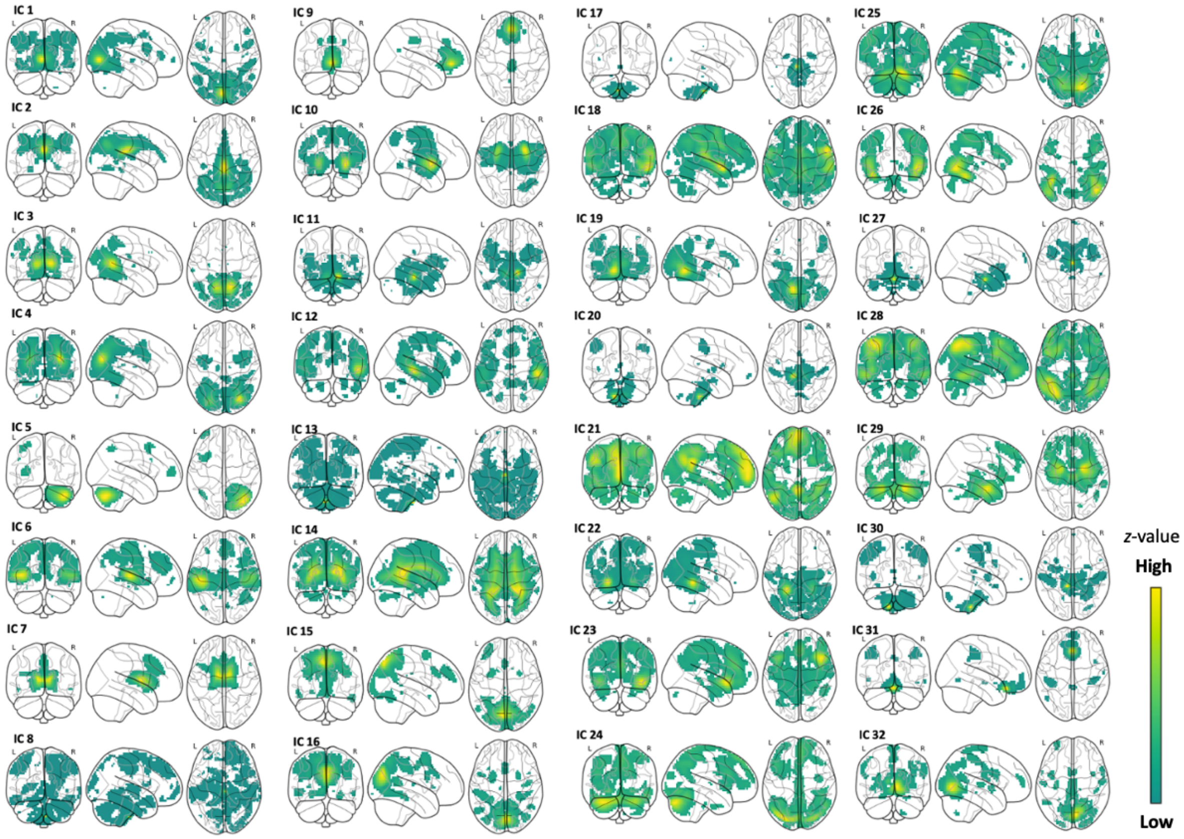
IC 1
IC 2
IC 3
IC 4
IC 5
IC 6
IC 7
IC 8
IC 9
IC 10
IC 11
IC 12
IC 13
IC 14
IC 15
IC 16
IC 17
IC 18
IC 19
IC 20
IC 21
IC 22
IC 23
IC 24
IC 25
IC 26
IC 27
IC 28
IC 29
IC 30
IC 31
IC 32
z-value
High
Low

(B)

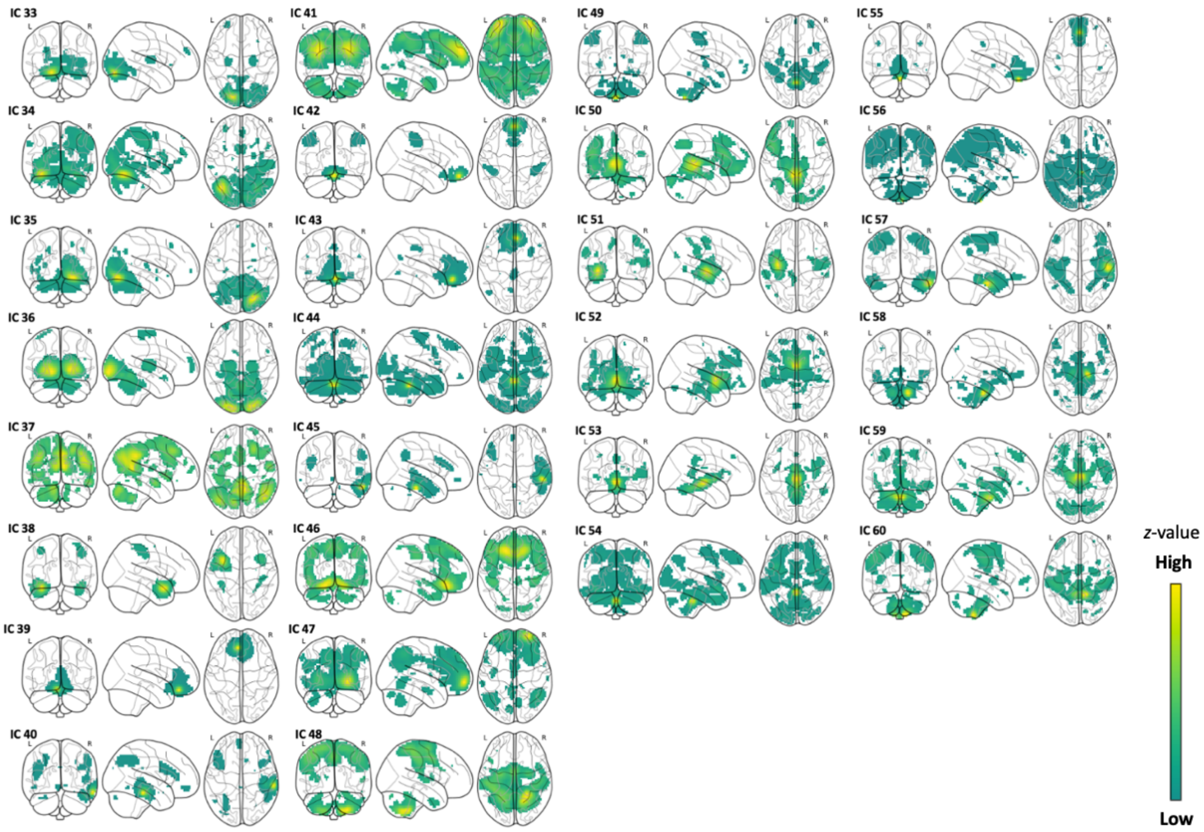

Fig. S11. The full set of ICs during picture encoding, in subsample 1. We used group-based ICA to decompose the functional data during picture encoding (unrelated to memory performance) into 60 ICs. ICs 1 to 32 and ICs 33 to 60 are illustrated in **(A)** and **(B)**, respectively.

(A)

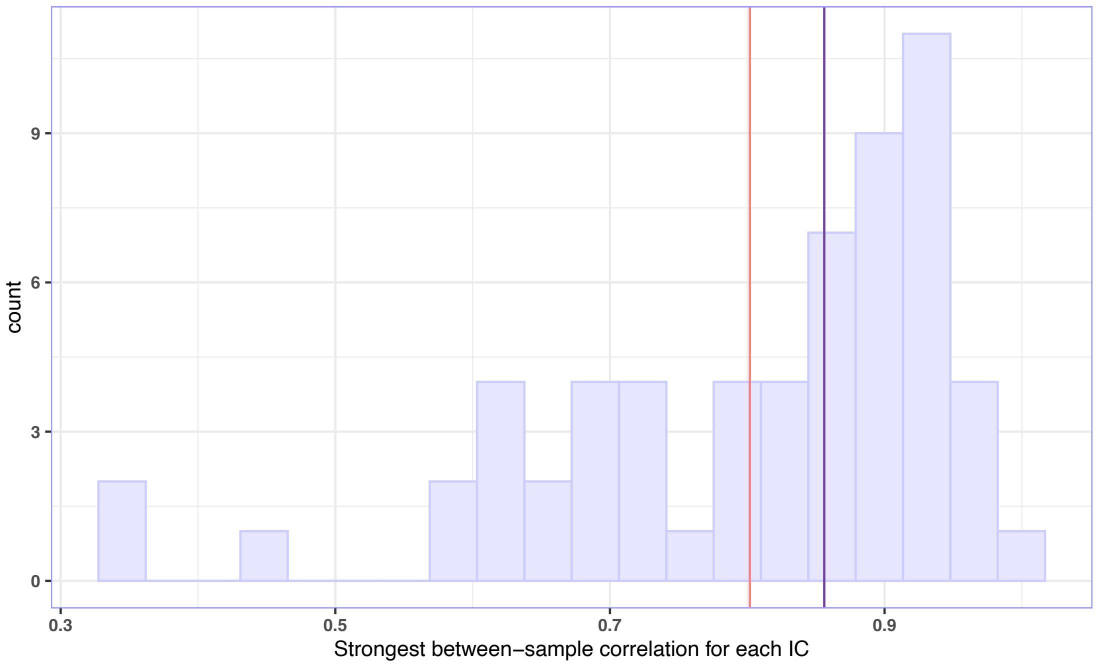

(B)

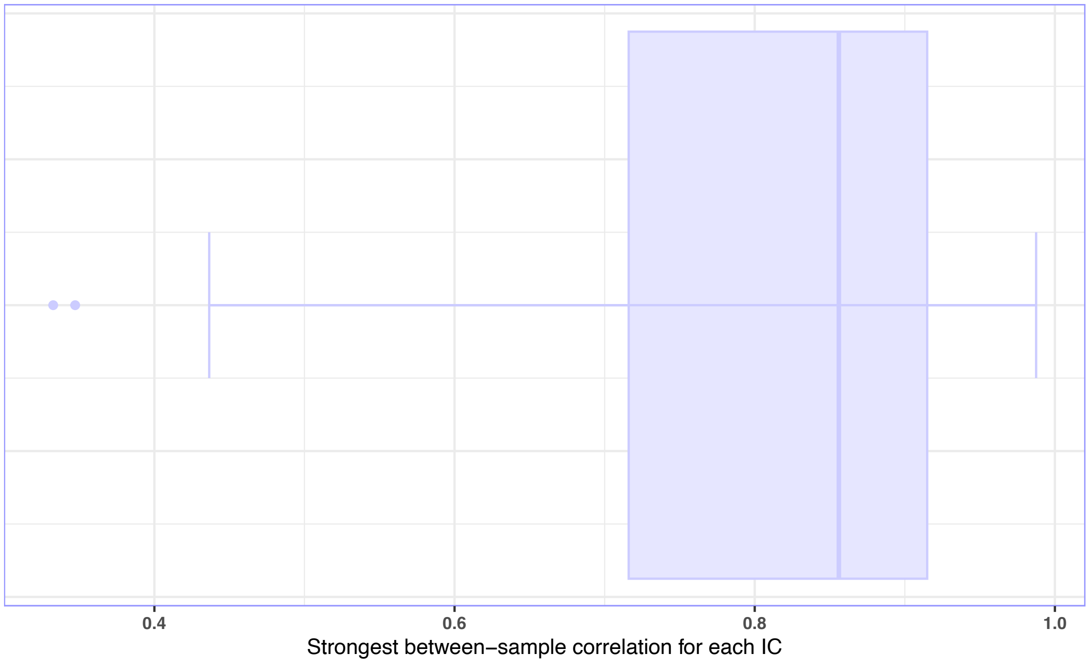

Fig. S12. Illustration of the cross-correlation values between the ICA solution in subsample 1 and subsample 2. Fifty out of 60 ICs surpassed $|r|_{max}$ >= 0.65, including all non-artefactual ICs with brain-behavior correlations. The distribution was left-skewed. The vertical lines in **(A)** denote the mean (blue) and median (red) with values of 0.856 and 0.802, respectively. The left and right whiskers in **(B)** extend from the hinge to the lowest and highest value within 1.5 times of the interquartile range, respectively. Source data are provided as a Source Data file.

(A)

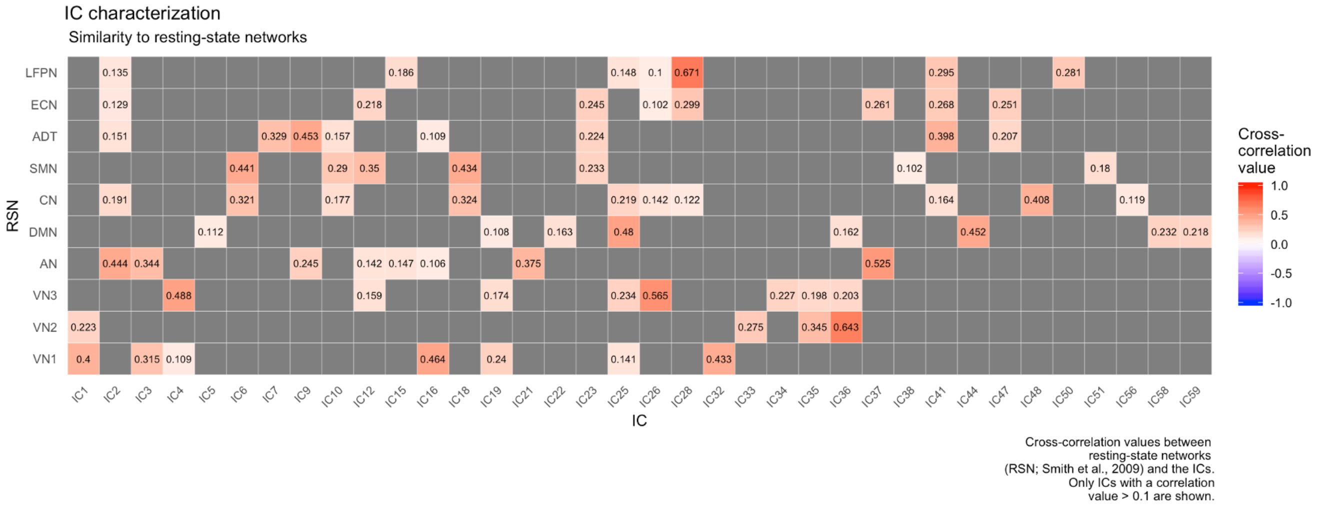

(B)

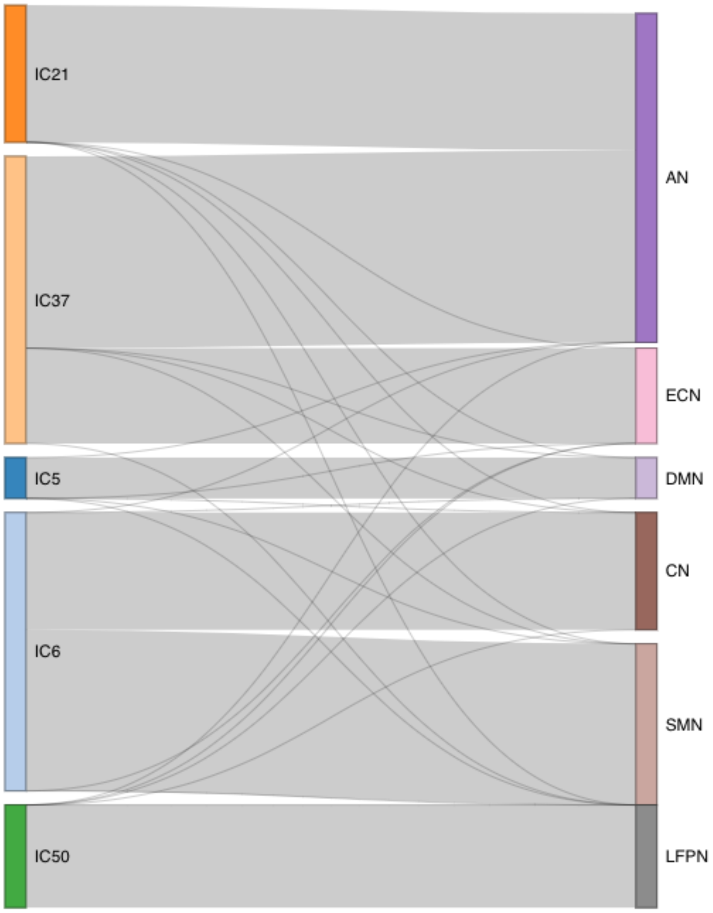

Fig. S13. Comparison of the ICs with resting-state networks. To characterize the ICs extracted from subsample 1 further, we compared their spatial appearance with that of taxonomic resting-state networks (see Methods), as illustrated in the **(A)** cross-correlation matrix, and the **(B)** Sankey plot, restricted to ICs associated with inter-individual differences in memory performance. VN = visual network; AN = attention network; DMN = default mode network; CN = cerebellar network; SMN = sensorimotor network; ADT = auditory network; ECN = executive control network; LFPN = left frontoparietal network. Source data are provided as a Source Data file.

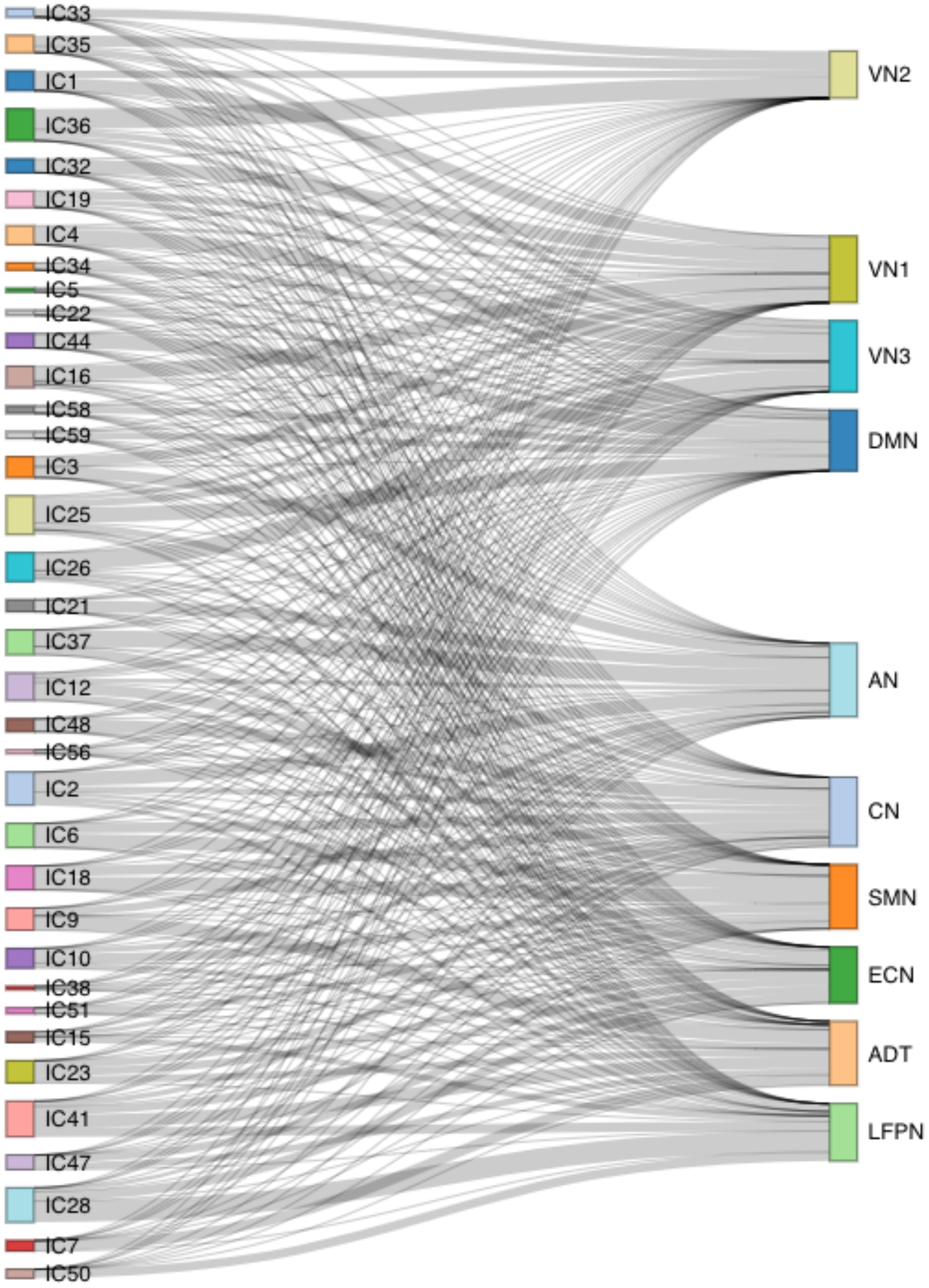

Fig. S14. Comparison of the ICs with resting-state networks. In the sankey plot only ICs with a cross-correlation value above 0.1 are shown. VN = visual network; AN = attention network; DMN = default mode network; CN = cerebellar network; SMN = sensorimotor network; ADT = auditory network; ECN = executive control network; LFPN = left frontoparietal network.

(A)

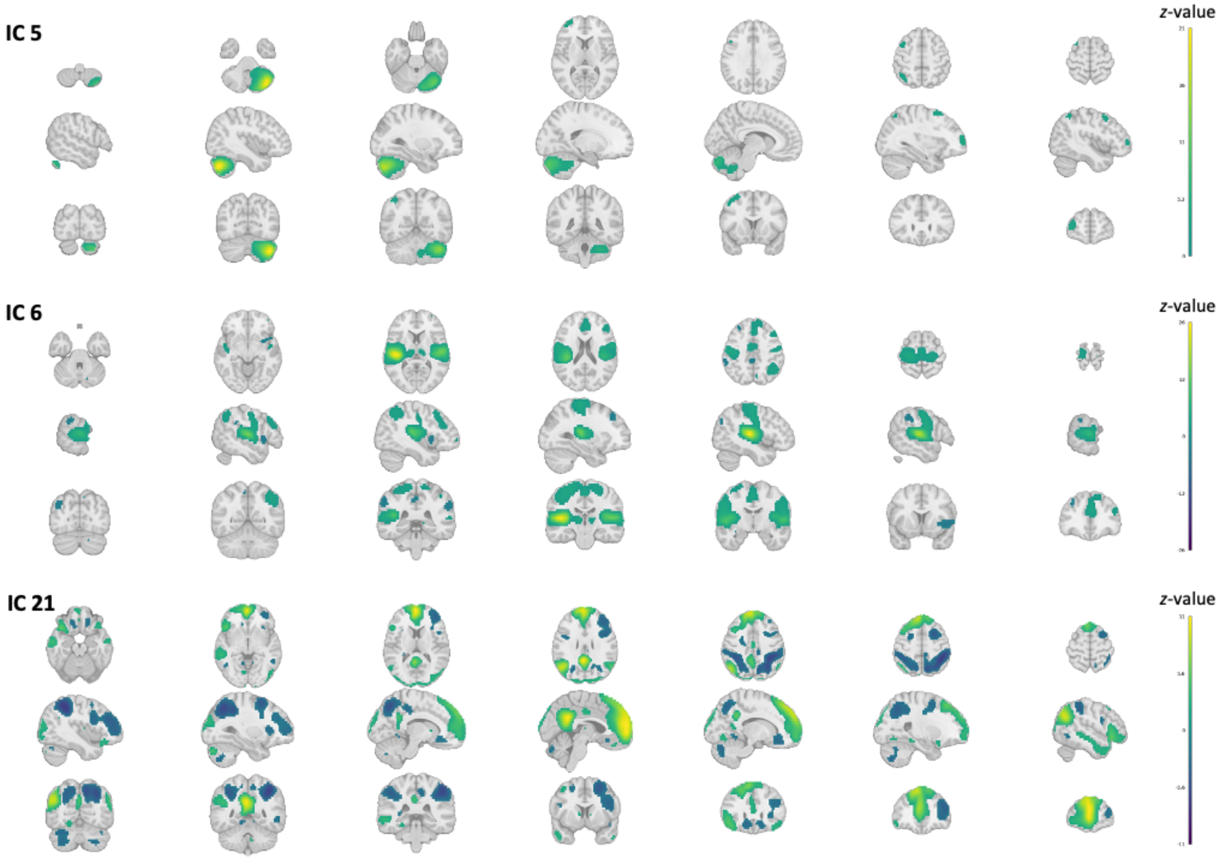

(B)

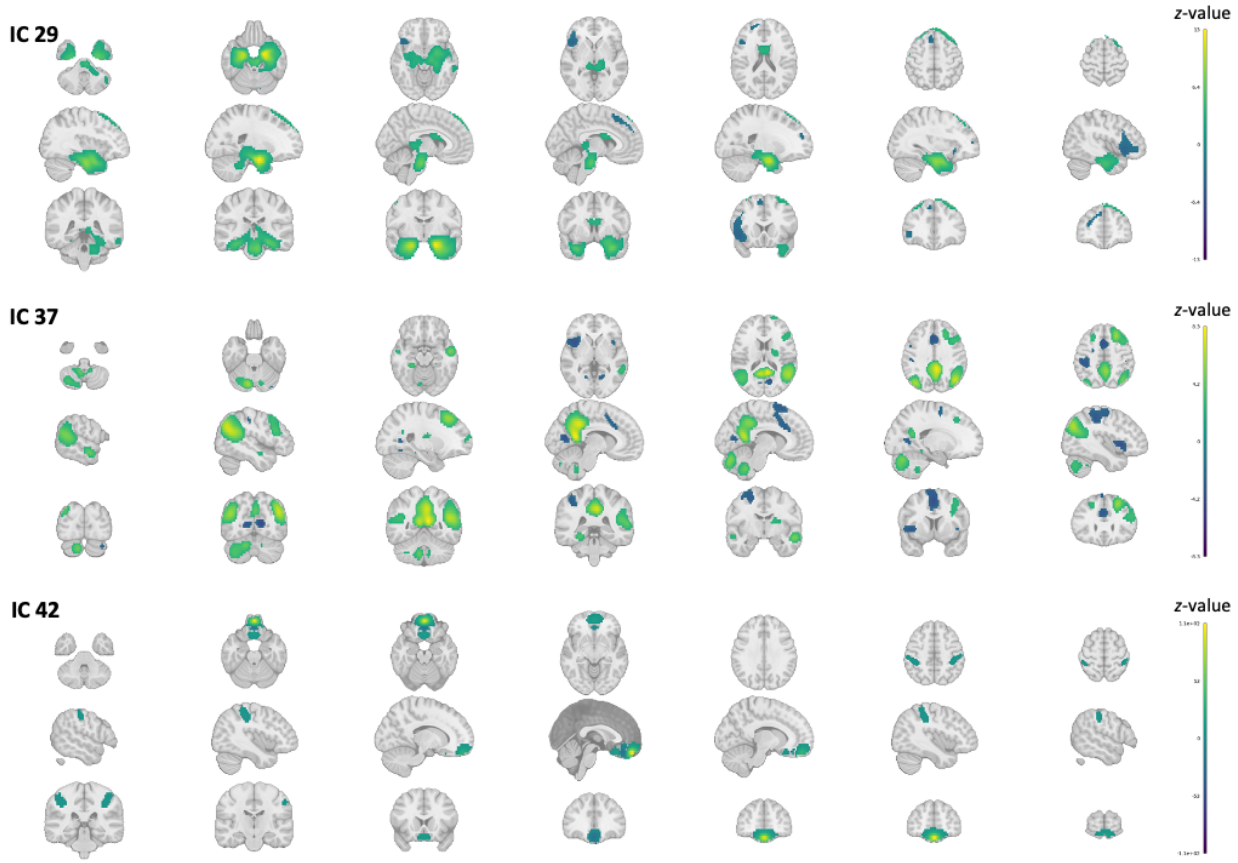

(C)

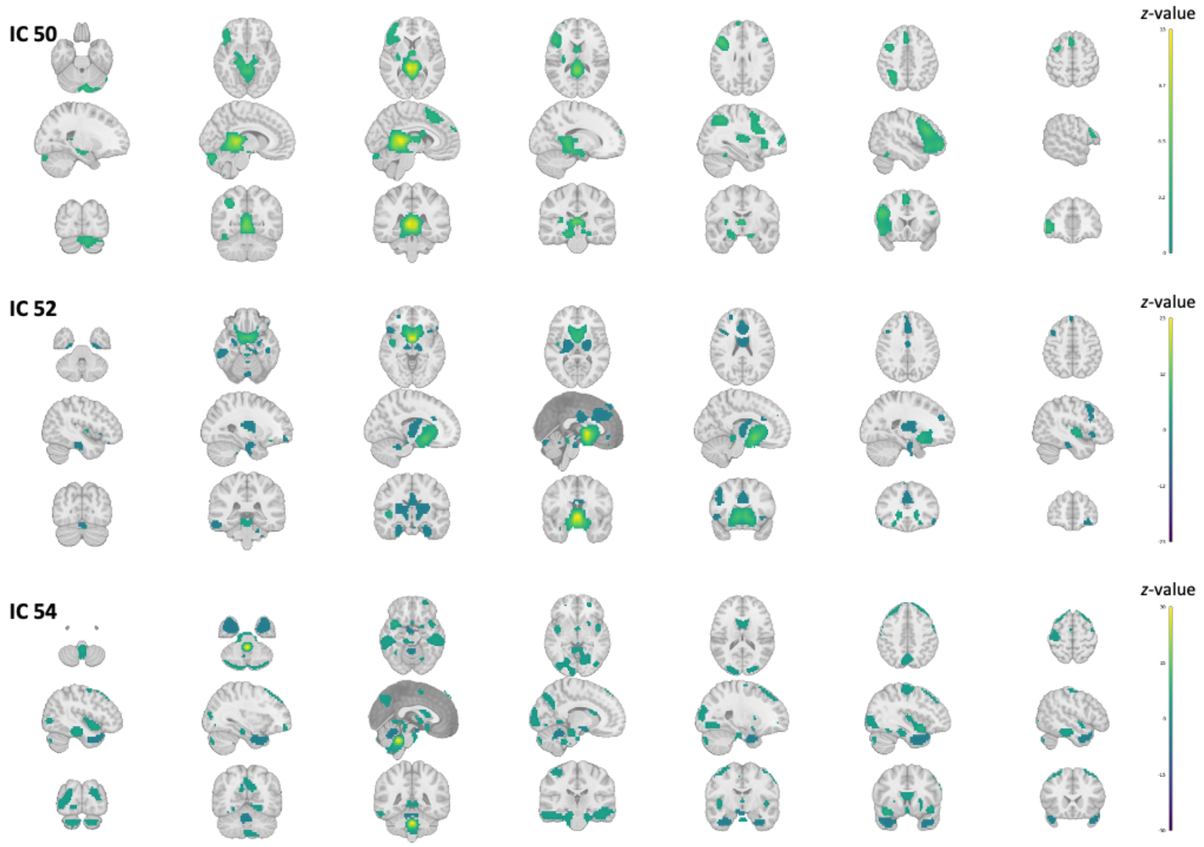

Fig. S15. The ICs with brain-behavior correlations. *Z*-values run along a spectrum from yellow to dark green, respectively, high to low values. See **(A)** for ICs 5, 6 and 21, **(B)** for ICs 29, 37 and 42, **(C)** for ICs 50, 52 and 54.

(A)

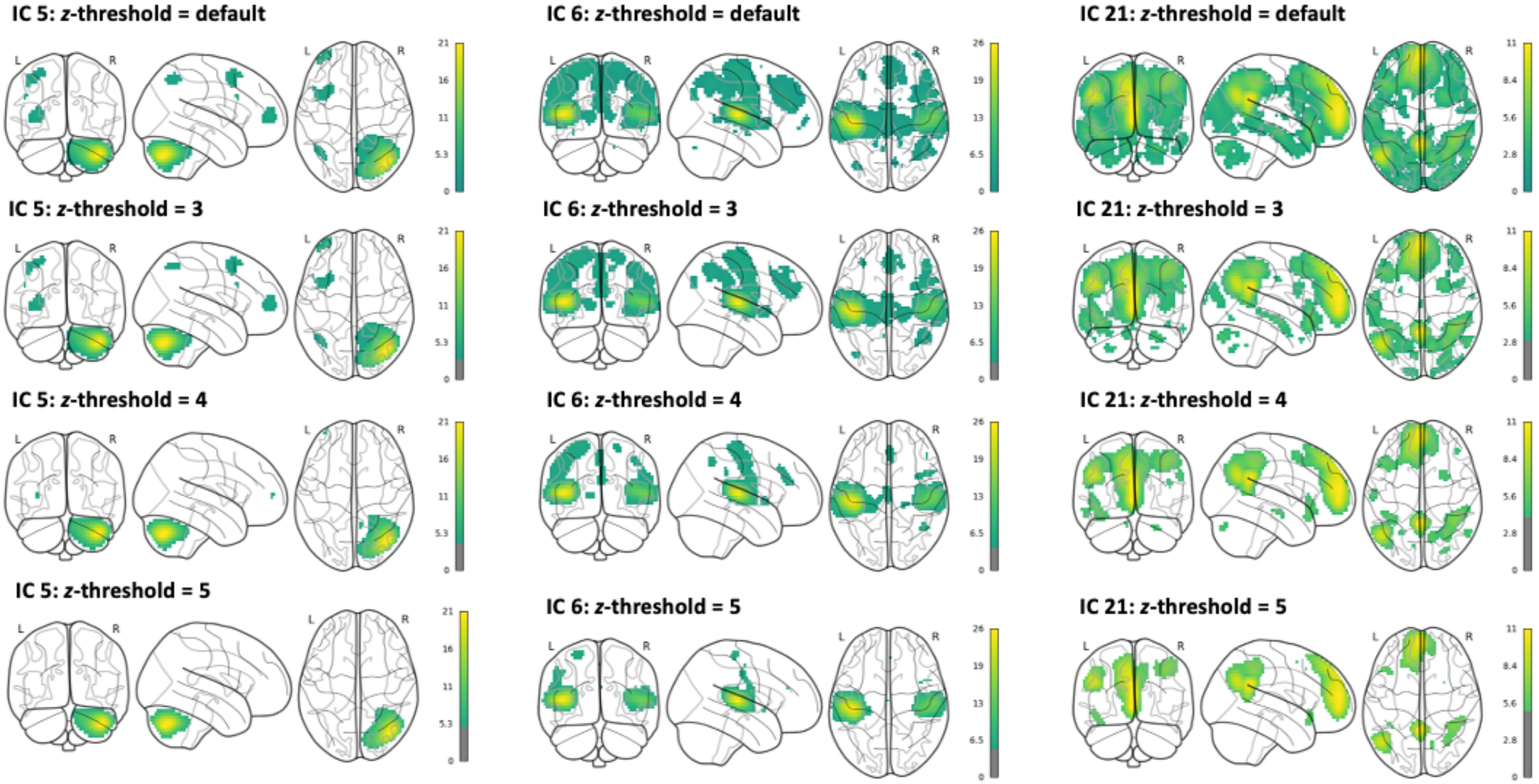

IC 5: z-threshold = default
IC 6: z-threshold = default
IC 21: z-threshold = default
IC 5: z-threshold = 3
IC 6: z-threshold = 3
IC 21: z-threshold = 3
IC 5: z-threshold = 4
IC 6: z-threshold = 4
IC 21: z-threshold = 4
IC 5: z-threshold = 5
IC 6: z-threshold = 5
IC 21: z-threshold = 5

(B)

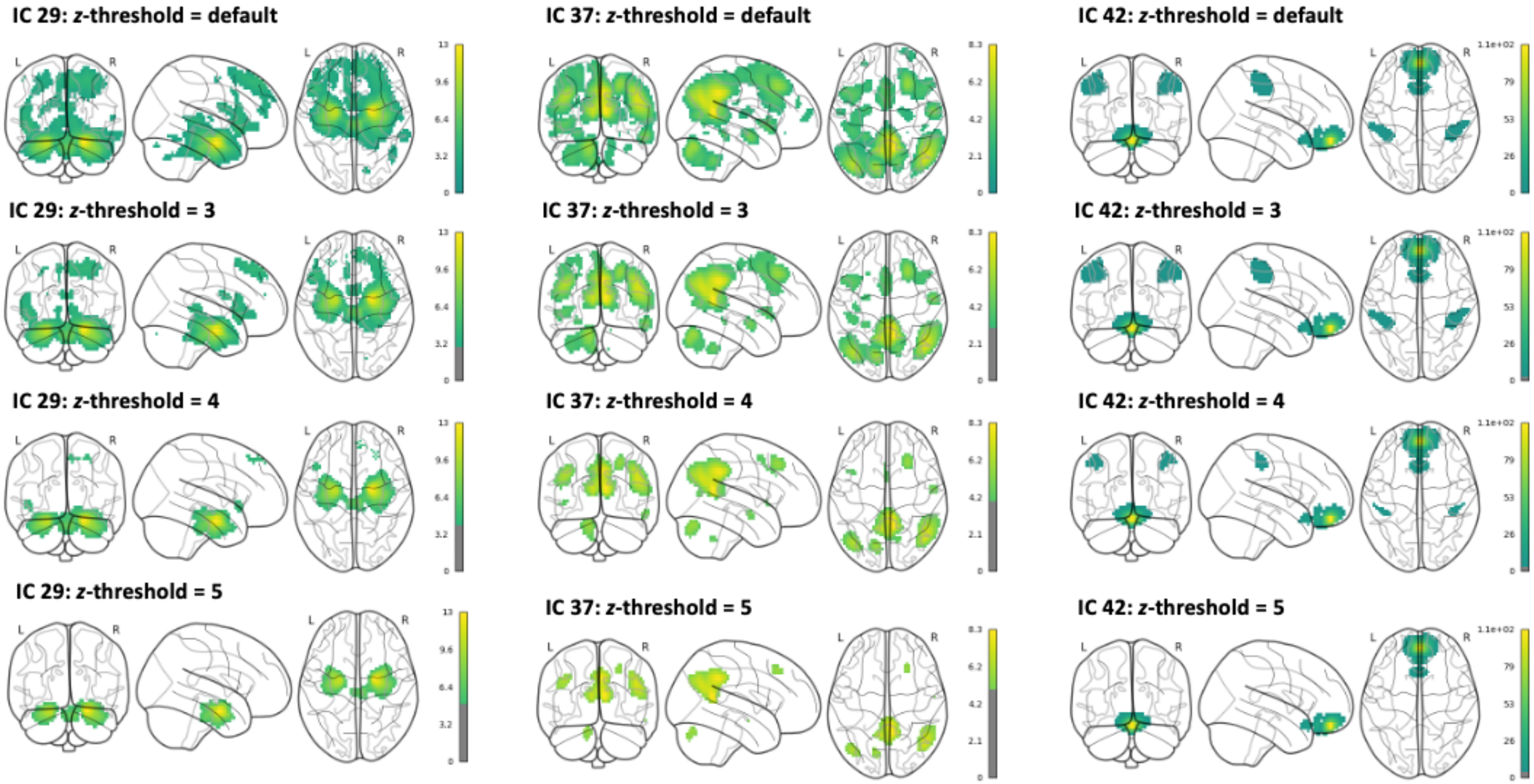
IC 29: z-threshold = default
IC 37: z-threshold = default
IC 42: z-threshold = default
IC 29: z-threshold = 3
IC 37: z-threshold = 3
IC 42: z-threshold = 3
IC 29: z-threshold = 4
IC 37: z-threshold = 4
IC 42: z-threshold = 4
IC 29: z-threshold = 5
IC 37: z-threshold = 5
IC 42: z-threshold = 5

(C)

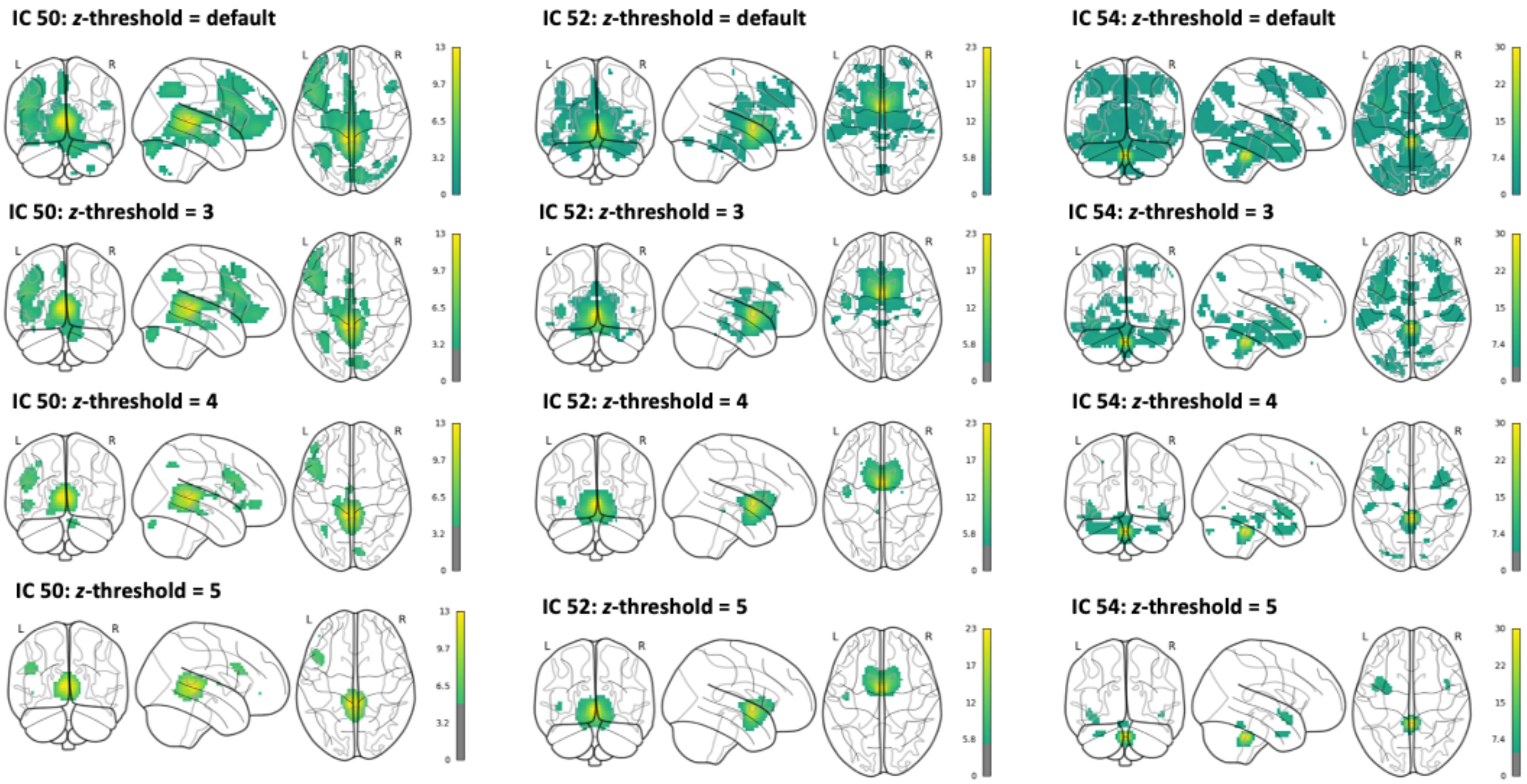

Fig. S16. Maps of ICs with different *z*-thresholds. ICs with different *z*-thresholds, namely FSL's MELODIC default in the top row of this illustration, followed by increasing *z*-values thresholds in the rows below (thresholds of |*z*-values| of 3, 4 and 5). The *z*-values inform on the contribution of each voxel to the making of an IC. The higher a voxel's *z*-value, the larger its contribution to an IC. See **(A)** for ICs 5, 6, 21, **(B)** for ICs 29, 37, 42, and **(C)** for ICs 50, 52, and 54.

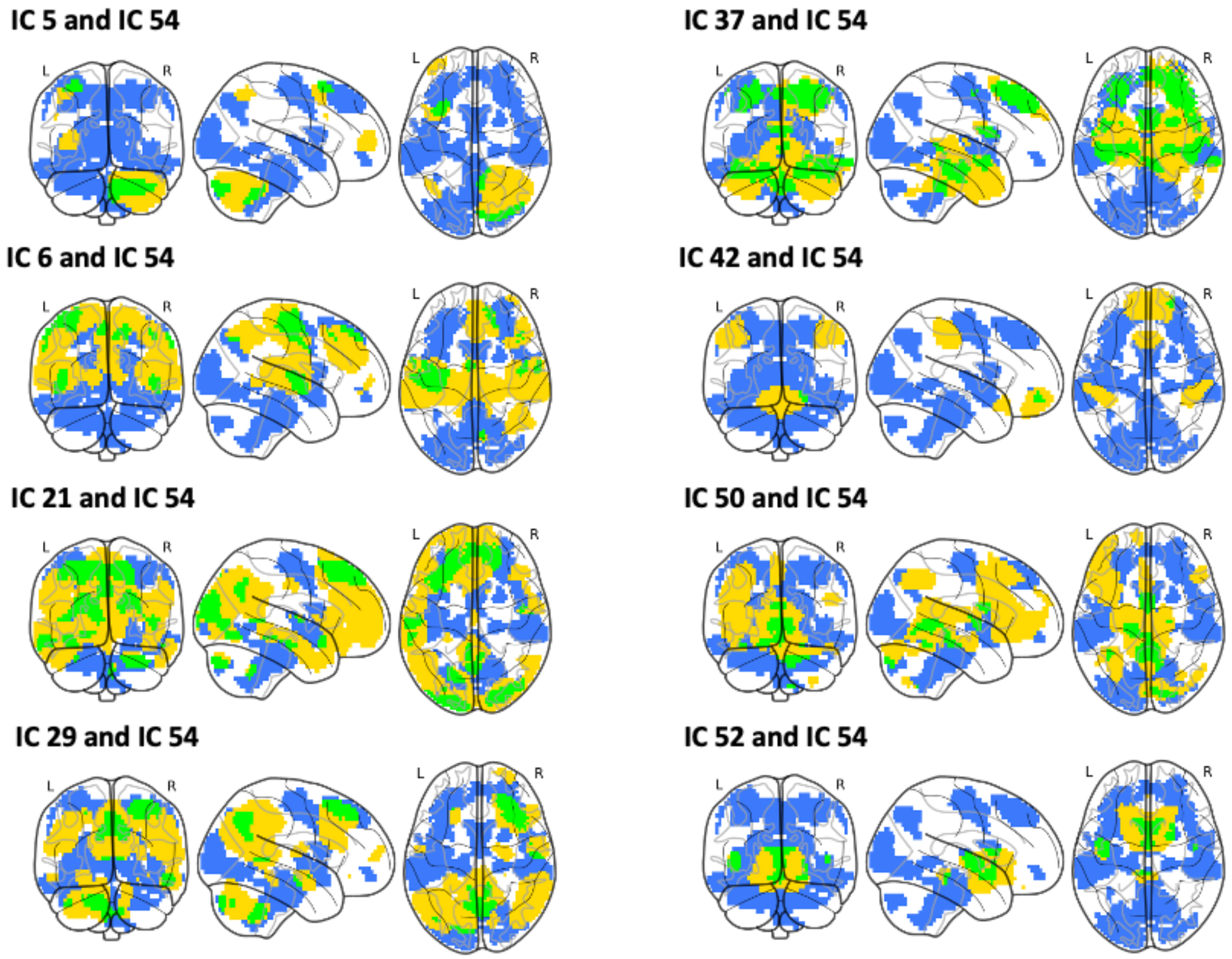

Fig. S17. Overlaps of IC 54 with each one of the other ICs with brain-behavior correlations. Color codes: blue, voxels unique to IC 54; yellow, voxels unique to the respective IC; green, overlapping voxels.

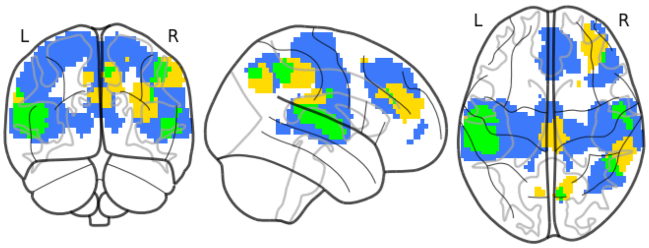

Fig. S18. Overlap of IC 6 and the memorability-controlled negative subsequent memory effects. IC 6 as the only IC with negative brain-behavior correlations demonstrated an overlap with the memorability-corrected negative subsequent memory effects. Color codes: yellow, voxels unique to the memorability-corrected negative subsequent memory effects; blue, voxels unique to IC 6; green, overlapping voxels.

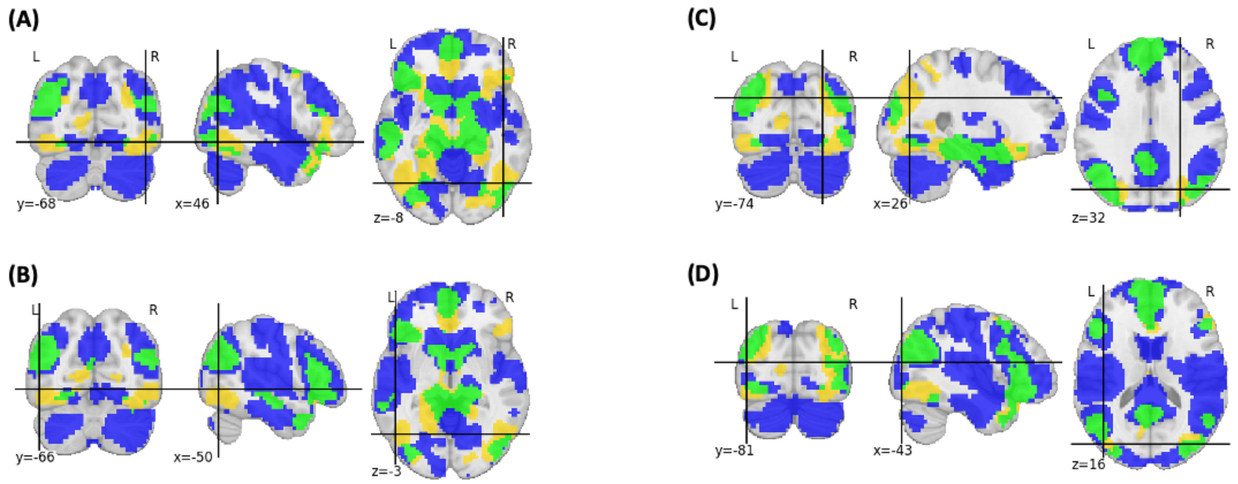

Fig. S19. Overlap of all ICs showing brain-behavior correlations and the memorability-corrected positive subsequent memory effects. (A) inferior lateral occipital cortex right **(B)** inferior lateral occipital cortex left **(C)** superior lateral occipital cortex right, **(D)** superior lateral occipital cortex left. Color codes: yellow, voxels unique to the memorability-corrected positive subsequent memory effects; blue, voxels unique to at least one of the ICs showing brain-behavior correlations; green, overlapping voxels.

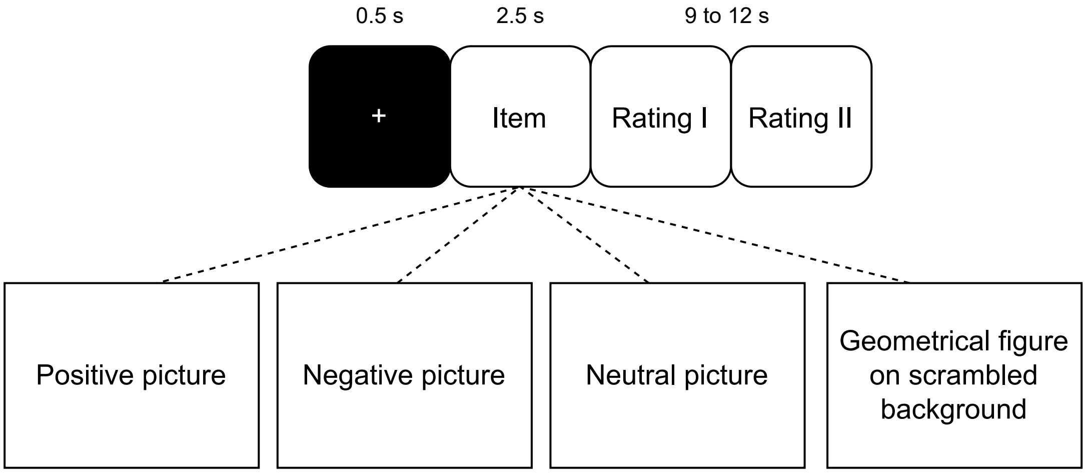

Fig. S20. Example trial of the encoding task. For each trial, the item displayed was either a positive, negative, or neutral picture, or a geometrical figure placed on a scrambled background. All subjects were exposed to every item category during the encoding task. Following the item presentation, subjects were asked to rate the item based on two sequential criteria. The combined duration for both ratings ranged from 9 and 12 s (jitter).

Dataset	Min (|*r*|)	Max (|*r*|)	Median (|*r*|)	*M* (|*r*|)	*SD* (|*r*|)
All 60 ICs	0.333	0.987	0.856	0.802	0.150
ICs with brain-behavior correlations	0.612	0.931	0.786	0.786	0.128

Table S1.

Descriptive statistics of the between-sample spatial correlations of the ICs. The table provides the descriptive statistics of the spatial correlation between ICs extracted from subsample 1 and subsample 2, as given for the complete set of 60 ICs and the ICs with brain-behavior correlations.

IC	Correlation value (|*r*|)	IC	Correlation value (|*r*|)
IC1	0.87	IC31	0.920
IC2	0.987	IC32	0.347
IC3	0.921	IC33	0.864
IC4	0.954	IC34	0.768
IC5	0.696	IC35	0.854
IC6	0.925	IC36	0.913
IC7	0.614	IC37	0.889
IC8	0.437	IC38	0.812
IC9	0.914	IC39	0.943
IC10	0.977	IC40	0.661
IC11	0.963	IC41	0.881
IC12	0.946	IC42	0.931
IC13	0.840	IC43	0.930
IC14	0.917	IC44	0.691
IC15	0.923	IC45	0.333
IC16	0.894	IC46	0.692
IC17	0.767	IC47	0.885
IC18	0.891	IC48	0.624
IC19	0.879	IC49	0.940
IC20	0.793	IC50	0.786
IC21	0.697	IC51	0.876
IC22	0.972	IC52	0.612
IC23	0.776	IC53	0.820
IC24	0.729	IC54	0.641
IC25	0.736	IC55	0.855
IC26	0.865	IC56	0.726
IC27	0.896	IC57	0.829
IC28	0.614	IC58	0.857
IC29	0.898	IC59	0.722
IC30	0.591	IC60	0.598

Table S2.

Between-sample spatial correlations of the ICs. For each IC obtained from subsample 1, the highest between-sample voxel correlations with the ICs extracted from subsample 2 are shown.

IC	Variance explained
5	0.037
6	0.036
21	0.058
29	0.040
37	0.036
42	0.037
50	0.047
52	0.036
54	0.035

Table S3.

Variance explained of the brain-behavior correlations of the network-based approach. For each IC with brain-behavior correlations, the variance explained by the respective IC is provided.

Voxel-based brain-behavior correlations (brain region)	Covered by IC(s)
Precuneus/posterior cingulate	21, 37, 50
Medial temporal lobe (left)	29, 50
Frontal pole, frontomedial cortex	21, 42
Medial temporal lobe (right)	29, 50

Table S4.

Comparison of the brain-behavior correlations of voxel-based and the network-based approaches. Overview of the voxel-based brain-behavior correlation's coverage by the ICs. Clusters with at least 20 voxels (two-sided p-FWE-corrected < 0.05) were considered, wherein the overlap was at least 20 voxels. The comparison was of descriptive nature.

Methods S1.

The effects of memorability on picture encoding. This group-level analysis considered the average activation for the memorability-PM regressor. The model included age, sex, and batch effects (two MR gradient changes, one MR software upgrade, one of two rooms in which subjects completed the free recall task) as additional regressors. Whole-brain two-sided FWE correction for multiple comparisons was applied at a threshold of $p < 0.05$, with a minimum cluster size of 20 voxels. Please see Fig. S6 and Fig. S7 for results.
