# Supplementary material for: Neurofunctional underpinnings of individual differences in visual episodic memory performance
Source: Nat Commun. 2023 Sep 14;14:5694. doi: 10.1038/s41467-023-41380-w (PMC10502056; doi:10.1038/s41467-023-41380-w)
Supplement: Supplementary file 3 — Reporting Summary [file 41467_2023_41380_MOESM3_ESM.pdf]

nature portfolio

Corresponding author(s): Geissmann, Léonie
de Quervain, Dominique JF

Last updated by author(s): 2023/08/10

Reporting Summary

Nature Portfolio wishes to improve the reproducibility of the work that we publish. This form provides structure for consistency and transparency in reporting. For further information on Nature Portfolio policies, see our Editorial Policies and the Editorial Policy Checklist.

Statistics

For all statistical analyses, confirm that the following items are present in the figure legend, table legend, main text, or Methods section.

n/a	Confirmed	
☐	☒	The exact sample size (*n*) for each experimental group/condition, given as a discrete number and unit of measurement
☐	☒	A statement on whether measurements were taken from distinct samples or whether the same sample was measured repeatedly
☐	☒	The statistical test(s) used AND whether they are one- or two-sided<br>*Only common tests should be described solely by name; describe more complex techniques in the Methods section.*
☐	☒	A description of all covariates tested
☐	☒	A description of any assumptions or corrections, such as tests of normality and adjustment for multiple comparisons
☐	☒	A full description of the statistical parameters including central tendency (e.g. means) or other basic estimates (e.g. regression coefficient) AND variation (e.g. standard deviation) or associated estimates of uncertainty (e.g. confidence intervals)
☐	☒	For null hypothesis testing, the test statistic (e.g. *F*, *t*, *r*) with confidence intervals, effect sizes, degrees of freedom and *P* value noted<br>*Give P values as exact values whenever suitable.*
☒	☐	For Bayesian analysis, information on the choice of priors and Markov chain Monte Carlo settings
☐	☒	For hierarchical and complex designs, identification of the appropriate level for tests and full reporting of outcomes
☐	☒	Estimates of effect sizes (e.g. Cohen's *d*, Pearson's *r*), indicating how they were calculated

Our web collection on statistics for biologists contains articles on many of the points above.

Software and code

Policy information about availability of computer code

Data collection	The software Presentation® (Neurobehavioral Systems, Inc., Berkeley, CA, www.neurobs.com) was used for the pictorial rating task. Subjective ratings of the pictures presented were collected via this software.<br><br>Please see other sections for MRI scanning specifications.
Data analysis	SPM12 v. 6685 (Statistical Parametric Mapping, Wellcome Trust Centre for Neuroimaging, London, UK; http://www.fil.ion.ucl.ac.uk/spm/) implemented in Matlab R2016b.<br><br>MRTools' GLM Flex Fast2 (https://habs.mgh.harvard.edu/researchers/data-tools/glm-flex-fast2/).<br><br>FreeSurfer (v. 4.5). Fischl, B. et al. Whole brain segmentation: automated labeling of neuroanatomical structures in the human brain. Neuron 33, 341–355 (2002).<br><br>FMRIB Software Library M.M. Jenkinson, C.F. Beckmann, T.E. Behrens, M.W. Woolrich, S.M. Smith. FSL. NeuroImage, 62:782-90, 2012 Jenkinson, C.F. Beckmann, T.E. Behrens, M.W. Woolrich, S.M. Smith. FSL. NeuroImage, 62:782-90, 2012<br><br>FSL MELODICS in FSL v.5.0.9<br><br>Dual regression in FSL v.5.0.9 L. Nickerson, S.M. Smith, D. Öngür, C.F. Beckmann. Using Dual Regression to Investigate Network Shape and Amplitude in Functional Connectivity Analyses. Front Neurosci. 2017; 11: 115. doi: 10.3389/fnins.2017.00115

R Core Team (2022). R: A language and environment for statistical computing. R Foundation for Statistical Computing, Vienna, Austria. URL https://www.R-project.org/.

Brain maps were created with Nilearn (v. 0.8.1; https://nilearn.github.io/stable/index.html).

Specific R packages: ggplot2 (v. 3.4.0), ggdist (v. 3.3.0), networkD3 (v. 0.4), nlme (v. 3.1-153), dplyr (v. 1.0.10), oro.nifti (v. 0.11.4)

For manuscripts utilizing custom algorithms or software that are central to the research but not yet described in published literature, software must be made available to editors and reviewers. We strongly encourage code deposition in a community repository (e.g. GitHub). See the Nature Portfolio guidelines for submitting code & software for further information.

Data

Policy information about availability of data

All manuscripts must include a data availability statement. This statement should provide the following information, where applicable:

- Accession codes, unique identifiers, or web links for publicly available datasets
- A description of any restrictions on data availability
- For clinical datasets or third party data, please ensure that the statement adheres to our policy

Data Availability
Source data are provided as a Source Data file.
The individual fMRI data generated in this study and necessary to reproduce the voxel-based and network-based results have been deposited in the Open Science Framework database under accession code https://osf.io/7nhsg. The individual pre-processed fMRI data are not publicly available due size limitations, but are available from the corresponding authors upon request.

The group-level statistical brain maps (subsequent memory effects, memorability-corrected subsequent memory effects, voxel-based brain behavior correlations of the encoding contrast, voxel-based brain behavior correlations of the subsequent memory effect contrast, functional connectivity networks with brain-behavior correlations, arousal-corrected subsequent memory effects, memorability effects) have been deposited on the NeuroVault database under the accession code http://neurovault.org/collections/14303/), and the full set of 60 ICs, as calculated from subsample 1, has been deposited on Figshare under the accession code 10.6084/m9.figshare.c.6679262.

Code Availability
The following publicly available software packages were used for preprocessing, analysis, and figure creation: Matlab (v. R2016b), SPM12 (v. 6685), MRTools' GLM Flex Fast 2, FreeSurfer (v. 4.5), the FMRIB Software Library, FSL MELODIC (v. 5.0.9), FSL dual regression (v. 5.0.9), Nilearn (v. 0.8.1), RStudio (2022), and the related R packages ggplot2 (v. 3.4.0), ggdist (v. 3.3.0), networkD3 (v. 0.4), nlme (v. 3.1-153), dplyr (v. 1.0.10), and oro.nifti (v. 0.11.4).

Research involving human participants, their data, or biological material

Policy information about studies with human participants or human data. See also policy information about sex, gender (identity/presentation), and sexual orientation and race, ethnicity and racism.

Reporting on sex and gender	The sample consists of individuals of all sexes. Sex/gender were determined by self-report. We did not perform sex-specific or gender-specific subanalyses. We used self-reported sex as a covariate due to its known effects on the fMRI measures.
Reporting on race, ethnicity, or other socially relevant groupings	We did not classify subjects regarding variables such as race, ethnicity or other socially relevant groupings.
Population characteristics	The participants were healthy young individuals of all sexes (complete data n=1498; 930 females), aged 18 to 35 (25th percentile = 20, 75th percentile = 24; M = 22.44, SD = 3.31), The subjects were free of any lifetime neurological or psychiatric illness, and did not take any medication at the time of the experiment (except hormonal contraceptives).<br>Covariates were: Age, sex, MRI scanner update (scanner batch effects), room for behavioral tasks (location batch effects)<br>Distribution of the batch variables was as follows:<br>- Scanner gradient batch effects: three categories, of sample sizes 105, 264 and 1129<br>- Scanner software batch effects: two categories, of sample sizes 283 and 1215<br>- Free recall task room effects: three categories, of sample sizes 705, 266 and 527<br><br>For the network-based brain-behavior correlation analysis, 13 subjects were excluded (see below, information included in the main manuscript). This sample consisted of 567 males and 918 females, aged 18 to 35 years (M=22.45, SD=3.32). Distribution of batch variables was as follows:<br>- Scanner gradient batch effects: three categories, of sample sizes 103, 259 and 1123<br>- Scanner software batch effects: two categories, of sample sizes 278 and 1207<br>- Free recall task room effects: three categories, of sample sizes 694, 265 and 526

Recruitment	Advertising was done mainly at the University of Basel, Switzerland, and in local newspapers. We do not assume presence of self-selection biases.
Ethics oversight	Ethics committee of the Canton of Basel, Switzerland

Note that full information on the approval of the study protocol must also be provided in the manuscript.

Field-specific reporting

Please select the one below that is the best fit for your research. If you are not sure, read the appropriate sections before making your selection.

☐ Life sciences ☒ Behavioural & social sciences ☐ Ecological, evolutionary & environmental sciences

For a reference copy of the document with all sections, see nature.com/documents/nr-reporting-summary-flat.pdf

Behavioural & social sciences study design

All studies must disclose on these points even when the disclosure is negative.

Study description	One-group design, quantitative data, event-related fMRI design for contrast calculations.
Research sample	The age range was selected due to the known effects of aging on measures of the variables of interest (fMRI data, memory performance). The sample is representative for that age category.
Sampling strategy	In this large-scale fMRI study, a convenience sampling strategy was used. No prior sample size calculation was performed. For fMRI studies in particular, large sample sizes are important for the detectability, reliability and validity of statistical effects. Large sample sizes are preferrable for whole-brain analyses. This issue has further been emphasized with regards to brain-behavior correlation analyses (e.g., Dubois & Adolphs, 2016, Cerebral Cortex). The selection of the large sample size was particularly relevant because one of the main objectives of this study was to tackle issues emerging from small sample sizes. Along similar lines, the study aimed to verify effects that have previously been reported in studies where smaller sample sizes had been used.
Data collection	Neuroimaging data (structural, functional) was collected on an MRI scanner. Behavioral data (memory performance) was collected using paper-pencil. No one was present beside the researchers. The researchers were unaware of the research questions addressed in this study. There was only one study condition.
Timing	The Study went from May 2009 to March 2016.
Data exclusions	The same sample was used for all analyses with the exception of the network-based brain-behavior correlation analysis: of the full sample of 1,498 subjects, thirteen subjects were not included in the network-based brain-behavior correlation analysis because their data was not available at the time-point of data analysis.
Non-participation	This work is based on a Study where data collection had already been completed at the time-point of data analysis. Participants were excluded from the analyses based on the following criteria: 1) they did not fulfill the inclusion criteria; 2) they did not complete the whole task; 3) the quality of the structural and/or functional data was low; 4) data was not available at the time of analysis.
Randomization	There was one study group.

Reporting for specific materials, systems and methods

We require information from authors about some types of materials, experimental systems and methods used in many studies. Here, indicate whether each material, system or method listed is relevant to your study. If you are not sure if a list item applies to your research, read the appropriate section before selecting a response.

Materials & experimental systems

n/a	Involved in the study	
☒	☐	Antibodies
☒	☐	Eukaryotic cell lines
☒	☐	Palaeontology and archaeology
☒	☐	Animals and other organisms
☒	☐	Clinical data
☒	☐	Dual use research of concern
☒	☐	Plants

Methods

n/a	Involved in the study	
☒	☐	ChIP-seq
☒	☐	Flow cytometry
☐	☒	MRI-based neuroimaging

Magnetic resonance imaging

Experimental design

Design type	Event-related design
Design specifications	A session consisted in the presentation of 72 IAPS pictures from 3 valence categories (24 neutral, 24 negative, 24 positive), as well as 24 scrambled pictures, each depicting a geometrical figure. Two primacy and two recency pictures were presented at the beginning and the end the session, respectively. Those 4 pictures were not considered for the analyses. Pictures were presented for 2.5 s in a quasi-randomized order so that at maximum four pictures of the same category occurred consecutively. The encoding session lasted around 20 minutes.
Behavioral performance measures	Picture encoding (valence and arousal ratings for IAPS pictures, size and shape ratings for geometrical figures). This data was not used for analysis in the current study.<br>Free recall of IAPS pictures: paper-pencil description of pictures seen (an unannounced task)

Acquisition

Imaging type(s)	Structural (T1), functional (T2*)
Field strength	3 T
Sequence & imaging parameters	Measurements were performed on a Siemens Magnetom Verio 3 T whole-body MR unit equipped with a 12-channel head coil. Functional time series were acquired with a single-shot echo-planar sequence using parallel imaging (GRAPPA). We used the following acquisition parameters: TE (echo time) = 35 ms, FOV (field of view) = 22 cm, acquisition matrix = 80 × 80, interpolated to 128 × 128, voxel size: 2.75 × 2.75 × 4 mm3, GRAPPA acceleration factor r = 2.0. Using a midsaggital scout image, 32 contiguous axial slices placed along the anterior–posterior commissure plane covering the entire brain with a TR = 3000 ms (α = 82°) were acquired using an ascending interleaved sequence. The first two acquisitions were discarded due to T1 saturation effects. A high-resolution T1-weighted anatomical image was acquired using a magnetization prepared gradient echo sequence (MP-RAGE, TR = 2000 ms; TE = 3.37 ms; TI = 1000 ms; flip angle = 8; 176 slices; FOV = 256 mm; voxel size = 1 × 1 × 1 mm3).
Area of acquisition	A whole-brain scan was used

Diffusion MRI ☐ Used ☒ Not used

Preprocessing

Preprocessing software	fMRI data was preprocessed using SPM12 (Statistical Parametric Mapping, Wellcome Trust Centre for Neuroimaging; http://www.fil.ion.ucl.ac.uk/spm/) implemented in MATLAB R2016b (MathWorks).
Normalization	Normalization incorporated the following four steps: 1) Structural images of each subject were segmented using the 'New Segment' procedure in SPM12. 2) The resulting gray and white matter images were used to derive a study-specific group template.
Normalization template	The template was computed from a subgroup of 1.000 subjects, which were part of the subjects included in the present study. 3) An affine transformation was applied to map the group template to MNI space. 4) Subject-to-template and template-to-MNI transformations were combined to map the functional images to MNI space. The functional images were smoothed with an isotropic 8 mm full-width at half-maximum (FWHM) Gaussian filter.
Noise and artifact removal	Volumes were slice-time corrected to the first slice, realigned using the ‘register to mean’ option, and coregistred to the anatomical image by applying a normalized mutual information 3-D rigid-body transformation. Successful coregistration was visually verified for each subject. Subject-to-template normalization was done using DARTEL (51), which allows registration to both cortical and subcortical regions and has been shown to perform well in volume-based alignment (52). Normalization incorporated the following four steps: 1) Structural images of each subject were segmented using the 'New Segment' procedure in SPM12. 2) The resulting gray and white matter images were used to derive a study-specific group template. The template was computed from a subgroup of 1.000 subjects, which were part of the subjects included in the present study. 3) An affine transformation was applied to map the group template to MNI space. 4) Subject-to-template and template-to-MNI transformations were combined to map the functional images to MNI space. The functional images were smoothed with an isotropic 8 mm full-width at half-maximum (FWHM) Gaussian filter.<br>Normalized functional images were masked using information from their respective T1 anatomical file as follows. At first, the three-tissue classification probability maps of the “Segment” procedure (grey matter, white matter, and CSF) were summed to define the mask. The mask was binarized, dilated and eroded with a 3 × 3 × 3 voxels kernel using fslmaths (FSL) to fill in potential small holes. The previously computed DARTEL flowfield was used to normalize the brain mask to MNI space, at the spatial resolution of the functional images. The resulting non-binary mask was thresholded at 50% and applied to the normalized functional images. Consequently, the implicit intensity-based masking threshold usually employed to compute a brain mask from the functional data during the first level specification (spm_get_defaults('mask.thresh'), by default fixed at 0.8) was not needed any longer and set to a lower value of 0.05.
Volume censoring	Please see above the whole pre-processing description.

Statistical modeling & inference

Model type and settings	(i) Mass-univariate voxel-based subsequent memory effects<br>(ii) Mass-univariate voxel-based subsequent memory effects corrected for memorability<br>(iii) Mass-univariate voxel-based subsequent memory effects corrected for arousal<br>(iv) Brain-behavior correlations on voxel-level (correlation model of each voxel's single-subject standardized beta and free recall)<br>(v) resampling procedure of (iv) to test for its reproducibility<br>(vi) Brain-behavior correlations (correlation model of each IC's single-subject standardized beta and free recall)
Effect(s) tested	Contrasts of interest were as follows:<br>(i) Subsequent memory effects: "IAPS pictures later recalled - IAPS pictures later not recalled"<br>(ii) and (iii) involved the same as (i) whilst correcting for memorability and arousal, respectively<br>(vi) Network-based approach: Network responsivity: We used a brain-behavior correlation procedure. First, we computed network responsivity per IC for each subject (resulting in 60 values for each subject). These values were calculated as first-level standardized betas from the difference between IAPS pictures and geometrical figures estimates (standardized betas). Second, we implemented these in correlation models (brain-behavior correlation) with the other main variable of interest being numbers of pictures freely recalled. Covariates were included (see above).<br>(iv) Voxel responsivity: Brain-behavior correlations: The procedure was similar, except that instead of networks, voxels were used.<br>(v) We tested for reproducibility of (iv) by using a resampling procedure similar to Marek et al. (2022), where we tested the effects of using differently sized sample sizes on the robustness of the statistical values of these voxel-based brain-behavior correlations.

Specify type of analysis: ☒ Whole brain ☐ ROI-based ☐ Both

Statistic type for inference<br>(See Eklund et al. 2016)	Please see below.
Correction	Subsequent memory effects, subsequent memory effects corrected for arousal, subsequent memory effects corrected for memorability: A whole-brain FWE-correction was applied, with a significance threshold of $p < 0.05$<br><br>Brain-behavior correlation models:<br>In the case of the voxel-based approach, a whole-brain FWE-correction was applied, with a significance threshold of $p < 0.05$. The same correction was applied to the reproducibility analyses. In the case of the network-based approach, a Bonferroni correction was applied by dividing the statistical threshold by the number of ICs, resulting in a threshold of $p < 8.33e-04$ (0.05/60).

Models & analysis

n/a	Involved in the study
☒	☐ Functional and/or effective connectivity
☒	☐ Graph analysis
☒	☐ Multivariate modeling or predictive analysis
